# Novel DNA methylation changes in mouse lungs associated with chronic smoking

**DOI:** 10.1080/15592294.2024.2322386

**Published:** 2024-03-04

**Authors:** Chinonye Doris Onuzulu, Samantha Lee, Sujata Basu, Jeannette Comte, Yan Hai, Nikho Hizon, Shivam Chadha, Maria Shenna Fauni, Andrew J. Halayko, Christopher D. Pascoe, Meaghan J. Jones

**Affiliations:** a Department of Biochemistry and Medical Genetics, University of Manitoba, Winnipeg, Manitoba, Canada; b Biology of Breathing Theme, Children’s Hospital Research Institute of Manitoba, Winnipeg, Manitoba, Canada; c Department of Physiology and Pathophysiology, University of Manitoba, Winnipeg, Manitoba, Canada

**Keywords:** Chronic smoking, DNA methylation, lung function, epigenome-wide

## Abstract

Smoking is a potent cause of asthma exacerbations, chronic obstructive pulmonary disease (COPD) and many other health defects, and changes in DNA methylation (DNAm) have been identified as a potential link between smoking and these health outcomes. However, most studies of smoking and DNAm have been done using blood and other easily accessible tissues in humans, while evidence from more directly affected tissues such as the lungs is lacking. Here, we identified DNAm patterns in the lungs that are altered by smoking. We used an established mouse model to measure the effects of chronic smoke exposure first on lung phenotype immediately after smoking and then after a period of smoking cessation. Next, we determined whether our mouse model recapitulates previous DNAm patterns observed in smoking humans, specifically measuring DNAm at a candidate gene responsive to cigarette smoke, *Cyp1a1*. Finally, we carried out epigenome-wide DNAm analyses using the newly released Illumina mouse methylation microarrays. Our results recapitulate some of the phenotypes and DNAm patterns observed in human studies but reveal 32 differentially methylated genes specific to the lungs which have not been previously associated with smoking. The affected genes are associated with nicotine dependency, tumorigenesis and metastasis, immune cell dysfunction, lung function decline, and COPD. This research emphasizes the need to study CS-mediated DNAm signatures in directly affected tissues like the lungs, to fully understand mechanisms underlying CS-mediated health outcomes.

## Introduction

Smoking is one of the leading causes of preventable mortality worldwide, and poses a great burden on the healthcare system, as it has been linked to the development and poor clinical control of diseases like asthma, chronic obstructive pulmonary disease (COPD), bronchitis, obesity, and cancers [1–5]. Epigenetic mechanisms, particularly DNA methylation (DNAm), have been suggested as potential links between environmental exposures such as smoking and disease outcomes that might manifest later in life.

There is abundant evidence that smoking alters DNAm patterns in human peripheral blood [6–9] and leucocyte subtypes [7], lymphoblasts and pulmonary macrophages [10], and epithelial oral cells [11]. In fact, there are reports that methylation levels of certain CpGs are effective markers for predicting smoking status [12] and lung cancer incidence [13]. However, most of these past studies associating smoking with DNAm alterations were done in accessible tissues, such as blood or epithelia, where large-scale sample collections are possible. Data from large human studies using accessible tissues is extremely helpful in identifying potential biomarkers of exposure or outcome, but evidence from more proximal tissues such as lungs which could develop our mechanistic understanding is severely lacking [14]. DNAm is cell type and tissue-specific, and so it is not clear whether findings from peripheral tissues like blood can be applied to the lungs [15–18]. One recent study measured epigenome-wide DNAm in current smokers and reported that only a third of the significantly altered CpGs which they originally identified in whole blood were also altered in the lungs of current compared to never-smokers [14]. Another study compared smoking-induced DNAm changes in blood versus buccal cells and reported that DNAm sites from buccal cells outperformed those identified in blood cells in discrimination of 14 of 15 epithelial cancer types [19]. The latter study showed that tissues directly exposed to cigarette smoke (CS) such as lungs, buccal cells and saliva may demonstrate more similar effects of smoking on DNAm, but it is clear that for truly mechanistic understanding, it is essential to examine the main tissues of interest. It can be difficult to access these tissues in humans, so model organisms are often employed.

Studies using animal models to measure the effects of smoking on DNAm in the lungs are also limited [14,20,21]. One study measured the effect of 4 weeks of smoking on lung DNAm using capture-based bisulphite sequencing and found altered DNAm patterns for numerous genes, most involved in inflammation and in inflammatory injury in COPD [21]. Another measured epigenome-wide DNAm in the blood of current and never-smokers and then measured the corresponding DNAm at the significant CpGs in the lungs via pyrosequencing [14]. Their results revealed that only one-third significant CpGs in the blood also showed changes in DNAm in the lungs, demonstrating the importance of assessing DNAm in the specific tissue of interest [14]. A third study measured the effects of whole-body CS, tobacco carcinogens and lipopolysaccharides in mouse lungs using reduced representation bisulphite sequencing (RRBS) [20]. They reported DNAm and hydroxymethylation differences across multiple differentially methylated regions [20]. These studies are few and thus indicate that there is a clear need for more research on the effects of smoking on DNAm patterns in lung tissues.

DNAm microarray is a high-throughput method for epigenome-wide DNAm measurements at a lower cost and is less labour intensive than bisulphite sequencing. Sequentially increasing scales of the Infinium microarrays have been developed for human samples, but no such solution was available for use on mouse samples until recently, with the release of the Illumina Infinium mouse microarrays [22]. In the current study, we used an animal model of chronic smoking to measure effects of smoking on lung function, as well as gene expression and DNAm of a candidate gene, both immediately after smoking and 15 weeks after smoking cessation. We also employed the new mouse methylation array to measure epigenome-wide DNAm immediately after smoking. Our model successfully recapitulated some of the lung phenotypes and DNAm signatures observed in humans. It also reveals many novel sites in the lungs which are differentially methylated by smoking, involved in nicotine dependence, altered immune response and development of malignancies, lung function decline and COPD, cardiovascular defects and neurodegenerative disorders. This study is an essential step in understanding mechanisms underlying CS-induced health alterations, and therefore would help in developing therapies to mitigate the effects of smoking.

## Methods

### Animals and smoking model

This experiment was approved by the Animal Research Ethics and Compliance Committee of the University of Manitoba (protocol number 19–021 (AC11461)). Adult BALB/c mice (Charles River Laboratories, Massachusetts, United States) were given standard laboratory chow and clean water *ad libitum* and housed, four mice of a single sex per cage (except where noted), in individually ventilated cages in a 12-hour light/dark cycle throughout the duration of this experiment.

The full details of the smoke exposure paradigm have been published previously [23]. Briefly, 8-week-old adult female BALB/c mice were separated into two groups: 16 control mice and 16 smoke-exposed mice. Treatments were for a total period of 9 weeks, beginning 3 weeks prior to mating (at which point mice were singly housed), continuing throughout pregnancy and lasting until 3 weeks after the birth of offspring. Control mice were moved to a clean cage and exposed to room air, while CS mice were exposed to whole-body 1R6F research cigarettes (University of Kentucky, Lexington, KY), twice daily using the SCIREQ InExpose smoking robot (SCIREQ, Montreal, QC, Canada). The results reported in this study are a part of a larger experiment to investigate the effects of maternal smoking on offspring [23]. Therefore, the ‘dams’ referred to from this point forward were all pregnant in line with that experiment. Dams were weighed weekly and 48 to 72 hours after the last smoke exposure (referred to hereafter as ‘immediately after smoking’), a subset of dams underwent lung function testing followed by tissue collection. The remaining dams underwent lung function testing and tissue collection 15 to 16 weeks after smoking cessation (referred to hereafter as ‘15 weeks after smoking cessation’), resulting in data from 2 timepoints: immediately after 9 weeks of smoking and 15 weeks after smoking cessation (Sample and group information can be found in Supplementary Table S1). All supplemental information is available at: https://doi.org/10.6084/m9.figshare.24546013.v3.

### Measurement of maternal cotinine

To verify the delivery of CS, the cotinine levels were measured in plasma immediately after smoking, and again 15 weeks later in a subset of dams. Cotinine was measured using a commercially available ELISA kit (CalBiotech, Spring Valley, CA), according to the manufacturer’s instructions, and the concentration of cotinine in each sample was measured at 450 nm.

### Lung function measurement and Bronchoalveolar lavage fluid collection

Mice were anaesthetised with sodium pentobarbital and lung function measured using the SCREQ Flexivent small animal ventilator (SCIREQ Inc., Montreal, QC, Canada) as described previously [24,25]. Four lung function metrics: Total airway resistance (Rrs), Newtonian resistance (Rn), tissue resistance (G) and elastance (H) were assessed, first at baseline after injection of nebulized saline into the lungs, and then after introduction of increasing concentrations of nebulized methacholine (3 to 50 mg/mL). Methacholine serves as a cholinergic agonist which acts on muscarinic receptors in airway smooth muscles, thereby inducing bronchoconstriction and decreasing lung function [26,27]. As a result, methacholine challenge tests are considered the gold standard for measuring airway hyperresponsiveness in mouse models [26,27].

Once lung function measurements were complete, mouse lungs were washed twice with 1 mL of phosphate buffer saline (PBS) per wash, introduced via tracheal cannulation. The bronchoalveolar lavage fluid (BALF) obtained was centrifuged at 4°C at 1200 rpm for 10 min, and the supernatants were stored at −20°C to be used for future analysis. Cell pellets were resuspended in 1 mL of PBS for total cell counts using a haemocytometer. Differential cell counts of macrophages, eosinophils and lymphocytes were performed by first pipetting 100 uL of PBS-resuspended cell pellets onto glass slides using cytospin columns, staining with a modified Wright-Giemsa stain (Hema 3 Stat Pack), and then counting cells using a Carl Zeiss Axio Observer ZI microscope.

### Tissue collection, DNA/RNA isolation

We collected whole blood from dams immediately after 9 weeks of CS exposure and 15 weeks after smoking cessation. Blood obtained from the severed abdominal aorta was immediately pipetted into EDTA-coated tubes and centrifuged at 4000 rpm for 15 min to obtain plasma. Following separation, blood cell pellets and plasma were snap-frozen in dry ice and stored at −80°C. Next, left, middle, superior, inferior and postcaval lung lobes were collected into separate tubes and snap-frozen in dry ice.

To prepare for simultaneous DNA and RNA extraction using the Invitrogen DNA and RNA isolation kits, we homogenized whole left lungs in Qiagen RLT Plus buffer using the Qiagen Tissue Lyser II. We also extracted DNA from blood cell pellets using the Qiagen DNAeasy Blood and Tissue kit and quantified all extracted DNA and RNA using a NanoDrop spectrophotometer (NanoDrop Technologies, USA).

### Selection of candidate genes to measure DNAm in mice

As our mouse model was novel, we needed to confirm that we could use it to recapitulate some of the DNAm signatures previously reported in humans exposed to CS. We decided to measure DNAm at candidate genes first on a small scale and then proceed to epigenome-wide DNAm measurements. Complete details of how we selected our two candidate genes have been published previously [23]. Briefly, we selected two CpGs which were the most responsive to *in utero* CS in newborn umbilical cord blood samples as reported in a meta-analysis conducted across human cohorts, and had also been associated with exposure to CS in adulthood [28]. The two chosen human CpGs were *AHRR* (cg05575921) and *CYP1A1* (cg22549041). We then aligned these sequences against the GRCm38/mm10 *Mus musculus* genome assembly using muscle [29] alignment on R studio version 3.6. This produced mouse CpGs which exactly aligned with or were closest in position to the human CpGs selected from the meta-analysis. The two mouse CpGs were at chr13:74260517 for *Ahrr* and chr9:57696231 for *Cyp1a1* (GRCm38/mm10). It is essential to note that this region of *Ahrr* is not conserved between humans and mice, and while we selected the closest mouse CpG, it may not be comparable to the human position.

### Gene expression measurement

200 ng of lung mRNA was converted to cDNA using Maxima cDNA synthesis Kit (Thermo Fisher Scientific, Inc.) following manufacturer’s protocol. We measured the relative expression of *Ahrr* and *Cyp1a1* in dam lungs using quantitative real-time RT-PCR (qPCR) performed on the QuantStudio 3 Real-Time PCR System (Thermo Fisher Scientific, Waltham, MA). *Ahrr* and *Cyp1a1* expression was quantified using the ΔΔ^Cq^ method [30,31] after normalizing against mean *β-actin* and *Eif2a* levels in the same sample. Samples were run in duplicates under cycling conditions that were recommended by the manufacturer, and the primer sequences used have been published previously [23].

### *Measurement of DNAm at the candidate genes*, Ahrr *and* Cyp1a1

We measured DNAm at the two chosen mouse candidate genes, *Ahrr* and *Cyp1a1*, via pyrosequencing, as previously described [23]. Briefly, we performed bisulphite conversion (Zymo Research) on 500 ng of DNA isolated from the left lungs or blood to generate bisulphite-converted DNA (bcDNA), following the manufacturer’s protocol. Converted DNA was amplified using primers and conditions published previously [23]. DNAm at candidate genes was measured in duplicates using the Qiagen Q48 pyrosequencer, alongside mouse control DNA of increasing methylation concentrations: 0%, 25%, 50%, 75%, and 100%.

### Measurement of epigenome-wide DNAm in dam lung autosomes

We used the Illumina Infinium Mouse Methylation BeadChip (Illumina, San Diego, CA, USA) to measure DNAm across the whole mouse epigenome in accordance to the manufacturer’s protocol. Briefly, 750 ng of genomic DNA was bisulphite converted as described above. Next, bisulphite converted samples were randomized, amplified, fragmented, hybridized onto the array chip and scanned according to the standard protocol.

We exported IDAT files into R and performed preprocessing using the SeSAMe package [32]. Preprocessing steps included removal of 18,920 of 296,070 probes mapping to the X, Y and mitochondrial chromosomes, calculation of detection *p* values per probe using pOOBAH [32], background subtraction using noob [33], dye bias correction using the dyeBiasCorrTypeINorm function in the SeSAMe package and filtering out probes with detection *p* > 0.05. Next, signal intensities of the remaining 208,606 probes were quantified as β-values and used in downstream analyses.

### Measurement of epigenome-wide DNAm in dam lung X chromosomes

We measured the effect of smoking for 9 weeks on DNAm in Illumina mouse microarray probes mapping to the female X chromosomes separately. Similar to autosomal probes, preprocessing was conducted using the SeSAMe package in R [32]. We performed the preprocessing steps highlighted above on 15,117 of 296,070 probes mapping to the female X chromosome, and were left with 11,529 probes used in downstream analyses.

### Statistical analyses

Statistical analyses were performed in RStudio (versions 3.6.1 and 4.2). To analyse candidate gene pyrosequencing data, we averaged DNAm at each CpG across duplicates and measured between-group differences using student t-tests. We used one-way ANOVA to analyse lung function and differential cell count data, followed by student t-tests for multiple comparisons between groups at each methacholine dose. We considered *p* values <0.05 as significant.

To analyse epigenome-wide DNAm microarray data, we first performed chip, run, row and column batch correction on β values using ComBat [34], included in the SVA package [35]. Next, we used the SVA package to capture technical covariates to be included in our linear regression model, and included the recommended one surrogate variable in our model. Next, we used multivariable linear regression contained within the limma R package to measure differential DNAm on the β values. Since our study design has a very small sample size (*N* = 3 control, *N* = 3 CS) and our *p* values would therefore not accurately reflect very small differences [36] in DNAm, we considered CpGs as significant at *p* < 10e-3 and an effect size >0.05. We produced figures in R using the *ggplot2* and *Gviz* packages.

### Gene ontology

To obtain biological significance from identified differentially methylated probes, we used the ClusterProfiler R package [37] (v3.16.1) for gene enrichment analysis. We considered pathways with FDR < 0.05 to be significantly enriched.

## Results

**Figure 1.**
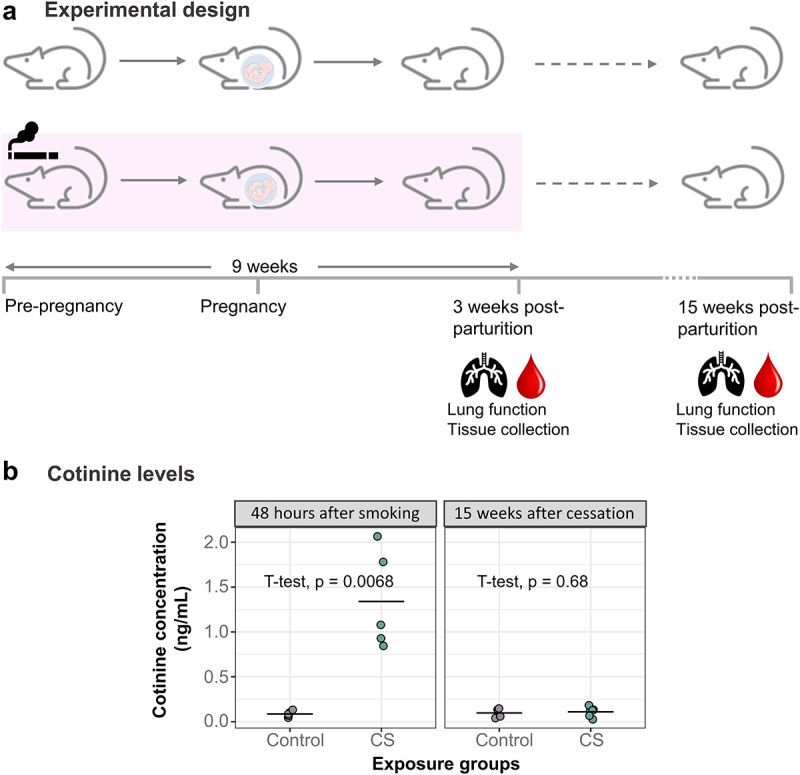
Development of a mouse model to study the effects of heavy smoking. (a) Adult female mice (*N* = 16 control, *N* = 16 CS) were exposed to heavy doses of whole-body CS for 9 weeks, from a pre-pregnancy period of 3 weeks and ending 3 weeks after birth of offspring. 15 of these had their litters within one week of each other and were included in this study. Following lung function measurements, tissues were collected from dams 48 hours after the last day of 9 weeks of smoke exposure and then 15 weeks after smoking cessation. (b) Dam cotinine levels measured in plasma 48 hours and 15 weeks after smoking cessation. *N* = 5 per group. Differences in plasma cotinine were analysed using student t-tests.

The research presented here is one part of a larger animal experiment to investigate the effects of prenatal CS exposure on offspring. We exposed BALB/c dams (*N* = 16 controls and *N* = 16 CS-exposed) to whole-body CS for a total period of 9 weeks, starting 3 weeks before mating, and lasting throughout pregnancy until 3 weeks after the birth of their pups (Figure 1(a)). CS-exposed dams tolerated smoking, with no losses or adverse events during the course of the experiments. We performed lung function and collected tissues for further analyses at two timepoints: 48–72 hours after the last day of 9 weeks of smoking (referred to hereafter as ‘immediately after smoking’), and 15–16 weeks after smoking cessation (referred to hereafter as ‘15 weeks after smoking cessation’) (Figure 1(a), Supplementary Table S1).

To verify absorption of CS components, we measured cotinine levels in plasma collected at these 2 different timepoints (Figure 1(b)). Dams which were exposed to CS had 15 times more elevated plasma cotinine levels (Mean = 1.34, SD = 0.55) immediately after smoking cessation compared to control dams (Mean = 0.09, SD = 0.03) (Figure 1(b), *p* = 0.0068). The relatively low levels of cotinine observed overall in the smoke-exposed dams can be attributed to the collection of plasma from dams approximately 48 hours after smoking, with the reported half-life of cotinine being about 24 hours [38,39]. Cotinine levels measured 15 weeks after CS exposure showed no significant difference between control and smoking dams (Figure 1(b)).

### Lung phenotype immediately after 9 weeks of smoking and 15 weeks after smoking cessation

Several studies have shown that personal smoking alters lung function and induces airway hyperresponsiveness [40–42]. Therefore, we assessed the effects of chronic smoking on the lung phenotype by measuring lung function and immune cell infiltration.

**Figure 2.**
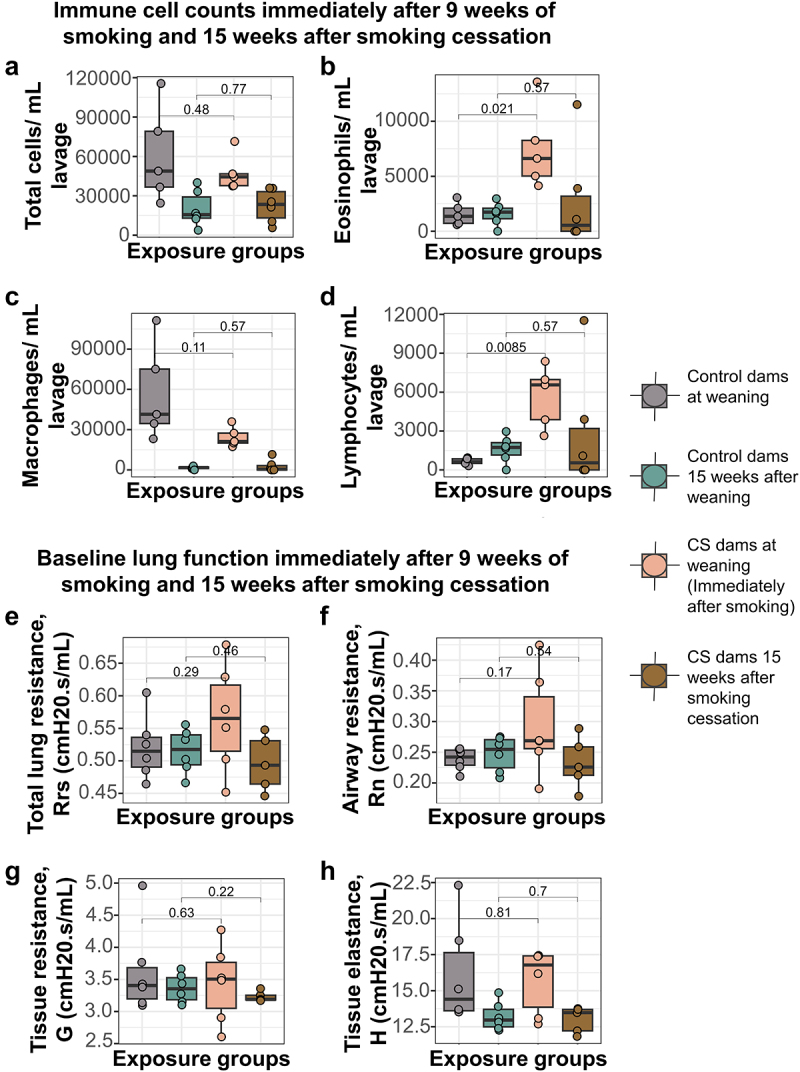
Smoke exposure causes transient changes to immune cell infiltration into dam lungs, but does not alter dam baseline lung function over time. (a) Total immune cells per mL lavage immediately after smoking and 15 weeks after smoking cessation. (b) Eosinophils per mL lavage in the CS-exposed dams (mean = 7528, SD = 3736) was over 4 times more elevated than control dams (mean = 1569, SD = 1029) immediately after smoking, but normalized to controls 15 weeks after smoking cessation. (c) Macrophages per mL lavage immediately after smoking and 15 weeks after smoking cessation. (d) Lymphocytes per mL lavage in the CS-exposed dams (mean = 5680, SD = 2357) was over 8 times elevated compared to control dams (mean = 649, SD = 256) immediately after smoking, but normalized to controls 15 weeks after smoking cessation. (e) Total lung resistance at baseline. (f) Airway resistance at baseline. (g) Tissue resistance at baseline. (h) Alveolar elastance at baseline. *N* = 4–6 per group. Differential cell counts were normalized to lavage volume. Lung function values were measured using 90^th^ percentile values after injection of saline into the lungs. Two-group comparisons were conducted using a student t-test and *p <* 0.05 was considered significant.

**Figure 3.**
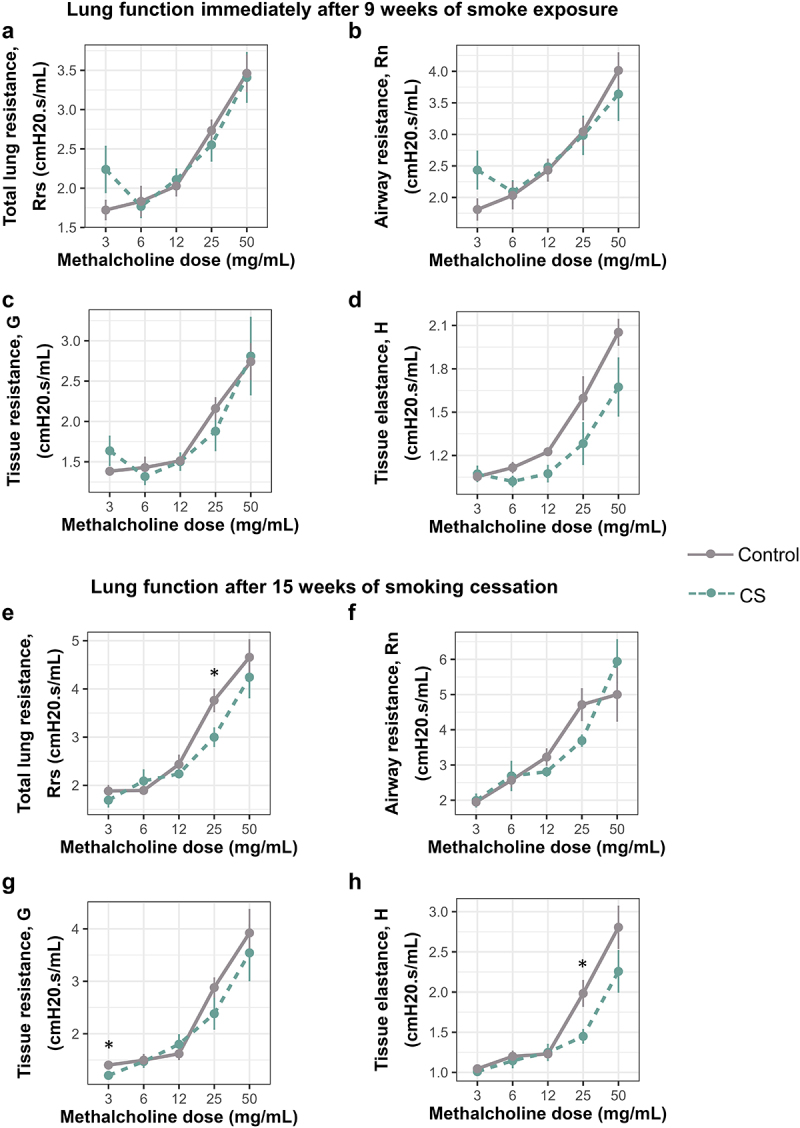
Smoke exposure alters methacholine responsiveness in mouse lungs up to 15 weeks after smoking cessation. (a) Total lung resistance immediately after smoking. (b) Airway resistance immediately after smoking. (c) Tissue resistance immediately after smoking. (d) Alveolar elastance immediately after smoking. (e) Total lung resistance after 15 weeks of smoking cessation. (f) Airway resistance after 15 weeks of smoking cessation. (g) Tissue resistance after 15 weeks of smoking cessation. (h) Alveolar elastance after 15 weeks of smoking cessation. *N* = 5–6 per group. Lung function values were measured using 90^th^ percentile values upon administration of increasing doses of methacholine. Data was analyzed using one-way ANOVA, followed by multiple comparisons at each methacholine dose where significant. *p < 0.05 in control vs smoke-exposed group.

Smoking for 9 weeks caused a fourfold and eightfold increase in eosinophils and lymphocytes, respectively, immediately after smoking, compared to controls (Figure 2(a–d)). The immune cell infiltration differences, however, did not persist until 15 weeks after smoking cessation (Figure 2(a–d)). Also of note is that total cell count and macrophage cell count were higher in both groups at the immediate post-smoking timepoint, when the dams were 3 weeks post-parturition and still lactating, than 15 weeks later (Figure 2(a,c)). We also found that direct CS exposure did not alter dam lung function either at baseline (Figure 2(e–h)) or after methacholine challenge after 9 weeks of smoking (Figure 3(a–d)), though tissue elastance was lower but not statistically significant in CS exposed than unexposed dams (Figure 3(d)). Thirteen weeks after smoking cessation, baseline lung function was also not altered (Figure 2(e–h)), but we observed significant differences between CS exposed and unexposed animals in tissue elastance, tissue resistance and total lung resistance values (Figure 3(e–h)).

Together, these data show that smoking for 9 weeks alters lung phenotype by increasing immune cell infiltration into the lungs immediately after smoking, accompanied by persistent decline in lung function even after smoking cessation.

### Candidate gene DNAm and expression immediately after 9 weeks of smoking and 15 weeks after smoking cessation

**Figure 4.**
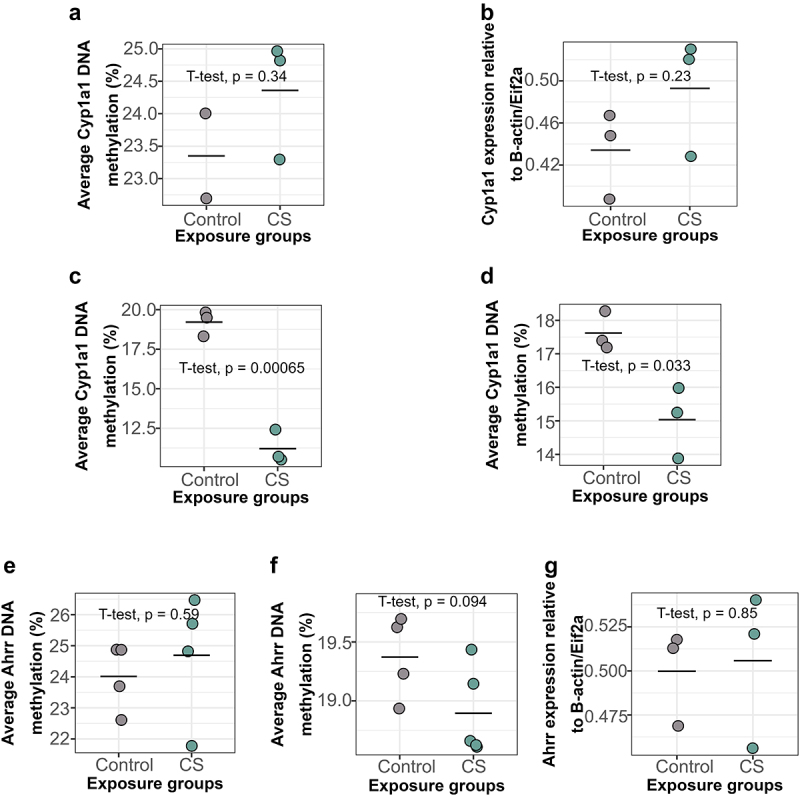
*Cyp1a1* and *Ahrr* DNAm and expression levels in dam blood and lungs immediately after smoking and 15 weeks after smoking cessation. (a) *Cyp1a1* DNAm in dam blood 15 weeks after smoking cessation. (b) *Cyp1a1* expression in dam lungs 15 weeks after smoking cessation. (c) *Cyp1a1* DNAm in dam lungs immediately after 9 weeks of smoking (d) *Cyp1a1* DNAm in dam lungs 15 weeks after smoking cessation. (e) *Ahrr* DNAm in dam blood after 15 weeks of smoking cessation. (f) *Ahrr* DNAm in dam lungs after 15 weeks of smoking cessation. (g) *Ahrr* expression in dam lungs after 15 weeks of smoking cessation. *N* = 2–5 per group. Differences in DNAm and expression were analysed using student t-tests.

To determine whether CS-induced DNAm changes observed in humans are recapitulated in our mouse model, we identified candidate genes to assess initially via pyrosequencing. We first measured DNAm and expression at *Cyp1a1* immediately after smoking and 15 weeks after cessation. We found an increase in *Cyp1a1* DNAm in the blood of CS-exposed dams 15 weeks after smoking cessation compared to controls, though this increase was not statistically significant (Figure 4(a)). *Cyp1a1* expression in the lungs of CS-exposed dams was slightly but not significantly elevated 15 weeks after smoking cessation (Figure 4(b)). Chronic exposure to CS for 9 weeks led to a significant decrease in *Cyp1a1* DNAm in the left lungs of CS-exposed dams immediately after smoking (Figure 4(c), Mean = 11.20, SD = 1.05), compared to controls (Figure 4(c), Mean = 19.20, SD = 0.80). Interestingly, *Cyp1a1* DNAm remained significantly decreased in the lungs of smoking dams (Figure 4(d), Mean = 15.00, SD = 1.07) compared to controls (Figure 4(d), Mean = 17.60, SD = 0.58) till 15 weeks after smoking cessation, though the magnitude had reduced slightly.

Conversely, we found no significant differences in *Ahrr* DNAm in dam blood 15 weeks after smoking cessation (Figure 4(e)). Similarly, *Ahrr* DNAm (Figure 4(f)) and *Ahrr* expression (Figure 4(g)) in dam lungs at this same timepoint was also not significantly different between groups. These results thus show that personal smoking for 9 weeks causes alterations in *Cyp1a1* DNAm in the lungs which persist for 15 weeks after smoking cessation.

### Measurement of effects of smoking on mouse lungs across the whole mouse epigenome using the Illumina mouse methylation microarrays

Following candidate gene DNAm measurements at *Ahrr* and *Cyp1a1*, we evaluated the association between smoking and epigenome-wide DNAm in the lungs immediately after 9 weeks of smoking, using normalized β values from the Illumina mouse microarrays. Due to our small sample size (*N* = 2–6 per group), we used a combination of *p* value (*p* < 1e-3) and effect size (0.05) cut-offs to determine significance. After adjusting for the one surrogate variable recommended by SVA (Supplementary Figure S1(a)), we performed multivariable regression on beta values from the autosomal probes (Supplementary Figure S1(a)) and identified 40 significant CpGs differentially methylated in dams exposed to heavy doses of CS for 9 weeks (Table 1). Maternal smoking in pregnancy led to higher DNAm at 37.5% (15 CpGs) and lower DNAm at 62.5% (25 CpGs) of these significant CpGs (Table 1). While our candidate gene, *Cyp1a1*, was not among our list of significant genes, a closer look at each of the *Cyp1a1* probes on the mouse array reveal large differences in DNAm between controls and CS-exposed dams in 3 of 9 *Cyp1a1* probes that passed preprocessing (Supplementary Figure S2, positions E, F and G). The second candidate gene, *Ahrr*, was also not significant in our epigenome-wide analysis and showed small effect size differences overall (Supplementary Figure S3).

**Table 1.** CpGs significantly altered immediately after exposure to CS for 9 weeks. Linear regression was computed using LIMMA, after adjusting for one surrogate variable recommended by SVA (see Figure S1).

CpG name	P value	Effect size	Direction	Gene	Chromosome	Strand	CpG position	Feature
cg28865575_TC11	0.000636	0.052702	Increase	Tmcc3	chr10	+	94565729	tss_body
cg29251423_BC21	0.000807	−0.06824	Decrease	Ddc	chr11	-	11849043	tss_body
cg29406381_BC11	0.000487	0.057487	Increase	Slit3	chr11	+	35415800	tss_body
cg29805460_BC21	0.000956	0.054231	Increase	Lyrm9	chr11	+	78837975	tss_body
cg30483569_TC21	0.000306	−0.07163	Decrease	Sox11	chr12	-	27337170	tss_body
cg30755159_TC21	0.000786	−0.05114	Decrease	Atl1	chr12	+	69922712	tss_body
cg30957340_TC21	0.000692	−0.05017	Decrease	Tshr	chr12	+	91500661	tss_body
cg31222899_BC21	0.000181	−0.06422	Decrease	Akr1c19	chr13	+	4233410	tss_1500
cg31361083_BC21	0.000395	−0.06943	Decrease	Mrs2	chr13	-	25018568	tss_body
cg31429252_TC21	0.00099	−0.05489	Decrease	NA	chr13	+	35067277	tss_body
cg32309765_TC21	0.000418	−0.06221	Decrease	Wnt5a	chr14	+	28520202	tss_body
cg34791132_BC11	0.000909	−0.06719	Decrease	Fkbp5	chr17	-	28420600	tss_body
cg34837962_BC21	0.000606	0.051306	Increase	NA	chr17	-	31905760	NA
cg35952853_BC21	0.000398	0.060032	Increase	Katnal2	chr18	-	77019702	tss_body
cg36020879_TC21	0.000498	−0.07337	Decrease	NA	chr18	+	83381494	NA
cg37170929_TC21	0.000902	−0.05191	Decrease	Sp100	chr1	+	85694662	tss_body
cg37188755_TC21	0.000491	0.064006	Increase	Snorc	chr1	+	87475345	tss_body
cg37214886_TC11	0.000816	0.058147	Increase	Asb18	chr1	-	89993031	tss_body
cg38811965_BC21	0.000584	−0.07976	Decrease	Aven	chr2	+	1.13E + 08	tss_body
cg39112318_BC11	0.000604	0.057822	Increase	NA	chr2	-	1.52E + 08	NA
cg39272146_BC11	0.00085	0.053982	Increase	NA	chr2	-	1.66E + 08	tss_1500
cg39843142_BC21	0.000369	−0.08598	Decrease	NA	chr3	-	66031662	tss_200
cg40162834_BC21	0.000984	−0.06193	Decrease	Strip1	chr3	-	1.08E + 08	tss_body
cg40351477_TC11	0.000227	−0.06665	Decrease	Npnt	chr3	-	1.33E + 08	tss_body
cg41660449_TC21	0.000465	−0.07261	Decrease	Aadacl3	chr4	-	1.44E + 08	tss_body
cg41773149_BC21	0.000755	−0.0517	Decrease	Megf6	chr4	+	1.54E + 08	tss_body
cg41898551_BC21	0.000703	−0.05057	Decrease	Magi2	chr5	+	20228413	tss_body
cg42734289_BC21	0.000476	0.070828	Increase	Atxn2	chr5	+	1.22E + 08	tss_body
cg42831686_BC11	0.000298	0.061434	Increase	NA	chr5	-	1.3E + 08	NA
cg43672977_TC21	0.000115	−0.07605	Decrease	Eefsec	chr6	-	88299565	tss_body
cg43685030_TC21	0.000655	−0.05455	Decrease	Chchd6	chr6	-	89496798	tss_body
cg44396689_TC21	0.000662	−0.08133	Decrease	NA	chr7	+	30681712	NA
cg44775237_BC21	0.000229	−0.06595	Decrease	Furin	chr7	-	80405386	tss_body
cg44780944_TC11	0.000578	0.057629	Increase	NA	chr7	+	80880504	NA
cg44880684_BC21	0.000489	−0.06252	Decrease	Nars2	chr7	+	97034436	tss_body
cg45732545_BC11	0.000884	−0.06968	Decrease	Galntl6	chr8	-	57805818	tss_body
cg45830215_BC11	8.72E–05	0.08256	Increase	B3gnt3	chr8	-	71701592	tss_body
cg45830217_BC21	0.000449	0.062246	Increase	B3gnt3	chr8	-	71701624	tss_body
cg46292742_BC21	0.000316	0.056784	Increase	Fbxo31	chr8	-	1.22E + 08	tss_body
cg46811672_TC21	0.000653	−0.06822	Decrease	Hcn4	chr9	+	58833272	tss_body

Results are sorted by CpG name. Direction: direction of change in DNAm compared to controls. Gene: gene names as annotated by UCSC Genome browser. Strand: direction of gene on the chromosomal strand (‘+’ = forward, ‘-’ = reverse). Feature: gene region feature as annotated by UCSC Genome browser (tss_body = located within the gene body, tss_200 = located 0–200 bases upstream of transcriptional start site, tss_1500 = located 200–1500 bases upstream of transcriptional start site). *N* = 3 Control and *N* = 3 CS. CpG sites were considered significant at *p* < 1e-3 and effect size > 0.05.

We performed a similar analysis on probes from the X chromosome, and after adjusting for the 1 surrogate variable recommended by SVA, found no significantly altered CpGs in dam lungs immediately after smoking (Supplementary Figure S4).

### Gene Ontology terms associated with smoking in the lungs

To gain insights into biological processes potentially affected by smoking in the lungs, we performed gene ontology analyses on genes mapping to the identified 40 significant CpGs. Gene ontology analysis revealed 54 biological processes which passed the FDR cut-off of 0.05 (Table 2). The top pathway findings were mostly driven by *Sox11, Wnt5a, Npnt, Slit3, Magi2* and *Furin*, all being involved in numerous processes. The most significantly implicated pathway with the smallest FDR (FDR = 0.007) was involved in regulation of transmembrane receptor protein serine/threonine kinase signalling pathways.

**Table 2.** List of significantly enriched biological pathways/processes immediately after 9 weeks of smoking.

ID	Description	P value	FDR	Gene ID	Gene count
GO:0090092	regulation of transmembrane receptor protein serine/threonine kinase signalling pathway	4.90E–06	0.007594	Sox11/Wnt5a/Npnt/Magi2/Furin	5
GO:0060412	ventricular septum morphogenesis	1.64E–05	0.012732	Slit3/Sox11/Wnt5a	3
GO:0009653	anatomical structure morphogenesis	2.86E–05	0.012997	Slit3/Sox11/Atl1/Tshr/Wnt5a/Sp100/Strip1/Npnt/Atxn2/Furin/Fbxo31	11
GO:0007178	transmembrane receptor protein serine/threonine kinase signalling pathway	3.36E–05	0.012997	Sox11/Wnt5a/Npnt/Magi2/Furin	5
GO:0031175	neuron projection development	6.38E–05	0.01459	Slit3/Atl1/Tshr/Wnt5a/Magi2/Atxn2/Fbxo31	7
GO:0060411	cardiac septum morphogenesis	6.79E–05	0.01459	Slit3/Sox11/Wnt5a	3
GO:0030182	neuron differentiation	6.97E–05	0.01459	Slit3/Sox11/Atl1/Tshr/Wnt5a/Magi2/Atxn2/Fbxo31	8
GO:0003281	ventricular septum development	7.54E–05	0.01459	Slit3/Sox11/Wnt5a	3
GO:0048699	generation of neurons	9.98E–05	0.017177	Slit3/Sox11/Atl1/Tshr/Wnt5a/Magi2/Atxn2/Fbxo31	8
GO:0048666	neuron development	0.000146	0.02261	Slit3/Atl1/Tshr/Wnt5a/Magi2/Atxn2/Fbxo31	7
GO:0071495	cellular response to endogenous stimulus	0.000173	0.024346	Sox11/Tshr/Wnt5a/Npnt/Magi2/Furin/Hcn4	7
GO:0090100	positive regulation of transmembrane receptor protein serine/threonine kinase signalling pathway	0.000194	0.024492	Sox11/Npnt/Furin	3
GO:0090287	regulation of cellular response to growth factor stimulus	0.000206	0.024492	Sox11/Wnt5a/Npnt/Furin	4
GO:0003279	cardiac septum development	0.000229	0.02522	Slit3/Sox11/Wnt5a	3
GO:0022008	neurogenesis	0.00025	0.02522	Slit3/Sox11/Atl1/Tshr/Wnt5a/Magi2/Atxn2/Fbxo31	8
GO:0040011	locomotion	0.00026	0.02522	Slit3/Tshr/Wnt5a/Sp100/Magi2/Furin/Fbxo31	7
GO:0062009	secondary palate development	0.00029	0.025719	Sox11/Wnt5a	2
GO:0090103	cochlea morphogenesis	0.000312	0.025719	Tshr/Wnt5a	2
GO:0071363	cellular response to growth factor stimulus	0.000328	0.025719	Sox11/Wnt5a/Npnt/Magi2/Furin	5
GO:0070848	response to growth factor	0.000361	0.025719	Sox11/Wnt5a/Npnt/Magi2/Furin	5
GO:0003206	cardiac chamber morphogenesis	0.000371	0.025719	Slit3/Sox11/Wnt5a	3
GO:0003231	cardiac ventricle development	0.000379	0.025719	Slit3/Sox11/Wnt5a	3
GO:0030511	positive regulation of transforming growth factor beta receptor signalling pathway	0.000409	0.025719	Npnt/Furin	2
GO:1903846	positive regulation of cellular response to transforming growth factor beta stimulus	0.000409	0.025719	Npnt/Furin	2
GO:0009719	response to endogenous stimulus	0.000432	0.025719	Sox11/Tshr/Wnt5a/Npnt/Magi2/Furin/Hcn4	7
GO:0040012	regulation of locomotion	0.000434	0.025719	Tshr/Wnt5a/Sp100/Magi2/Furin/Fbxo31	6
GO:0048812	neuron projection morphogenesis	0.000466	0.025719	Slit3/Atl1/Wnt5a/Atxn2/Fbxo31	5
GO:0120039	plasma membrane bounded cell projection morphogenesis	0.000513	0.025719	Slit3/Atl1/Wnt5a/Atxn2/Fbxo31	5
GO:0010922	positive regulation of phosphatase activity	0.000518	0.025719	Npnt/Magi2	2
GO:0048858	cell projection morphogenesis	0.000529	0.025719	Slit3/Atl1/Wnt5a/Atxn2/Fbxo31	5
GO:0048846	axon extension involved in axon guidance	0.000547	0.025719	Slit3/Wnt5a	2
GO:1902284	neuron projection extension involved in neuron projection guidance	0.000547	0.025719	Slit3/Wnt5a	2
GO:0000902	cell morphogenesis	0.000548	0.025719	Slit3/Atl1/Wnt5a/Strip1/Atxn2/Fbxo31	6
GO:0032990	cell part morphogenesis	0.000608	0.027706	Slit3/Atl1/Wnt5a/Atxn2/Fbxo31	5
GO:0120036	plasma membrane bounded cell projection organization	0.000629	0.027857	Slit3/Atl1/Tshr/Wnt5a/Magi2/Atxn2/Fbxo31	7
GO:0030030	cell projection organization	0.000731	0.030942	Slit3/Atl1/Tshr/Wnt5a/Magi2/Atxn2/Fbxo31	7
GO:0050919	negative chemotaxis	0.000739	0.030942	Slit3/Wnt5a	2
GO:0003205	cardiac chamber development	0.000829	0.033589	Slit3/Sox11/Wnt5a	3
GO:0071542	dopaminergic neuron differentiation	0.000846	0.033589	Tshr/Wnt5a	2
GO:0090102	cochlea development	0.000999	0.038672	Tshr/Wnt5a	2
GO:0032989	cellular component morphogenesis	0.001078	0.040397	Slit3/Atl1/Wnt5a/Atxn2/Fbxo31	5
GO:0043392	negative regulation of DNA binding	0.001121	0.040397	Sox11/Sp100	2
GO:0046189	phenol-containing compound biosynthetic process	0.001121	0.040397	Ddc/Wnt5a	2
GO:0048546	digestive tract morphogenesis	0.001207	0.042496	Sox11/Wnt5a	2
GO:0051239	regulation of multicellular organismal process	0.001241	0.042728	Sox11/Tshr/Wnt5a/Sp100/Npnt/Atxn2/Furin/Fbxo31/Hcn4	9
GO:0030100	regulation of endocytosis	0.001355	0.045622	Wnt5a/Magi2/Atxn2	3
GO:0048468	cell development	0.001461	0.047429	Slit3/Sox11/Atl1/Tshr/Wnt5a/Magi2/Atxn2/Fbxo31	8
GO:0002011	morphogenesis of an epithelial sheet	0.001531	0.047429	Sox11/Wnt5a	2
GO:0034340	response to type I interferon	0.001531	0.047429	Wnt5a/Sp100	2
GO:0045778	positive regulation of ossification	0.001531	0.047429	Sox11/Wnt5a	2
GO:0007399	nervous system development	0.001566	0.047577	Slit3/Sox11/Atl1/Tshr/Wnt5a/Magi2/Atxn2/Fbxo31	8
GO:0051049	regulation of transport	0.00167	0.049132	Sox11/Wnt5a/Sp100/Magi2/Atxn2/Furin/Hcn4	7
GO:0035306	positive regulation of dephosphorylation	0.001681	0.049132	Npnt/Magi2	2
GO:2000648	positive regulation of stem cell proliferation	0.001733	0.049701	Sox11/Wnt5a	2

ID: gene ontology identification code. Description: description of the enriched process. Gene ID: genes contributing to enrichment of the biological process. Gene count: number of genes contributing to the biological process. Biological processes were considered significantly enriched at FDR < 0.05.

## Discussion

Cigarette smoke is a complex mixture of over 7000 components [43–46], which affect virtually all body systems via mechanisms such as inflammation and DNA damage. While the processes linking smoking and adverse health outcomes are poorly understood, DNA methylation alterations have been identified as possible links due to their sensitivity to the environment and relative stability [47]. The association between smoking and changes in DNAm has been well documented, especially in human blood [6–9]. However, while there may be shared DNAm patterns between tissues [14,18], overall DNAm is highly variable across tissues [15–17], emphasizing the need to study smoke-induced DNAm alterations in more directly affected tissues such as the lungs. The major aim of this study, therefore, was to identify DNAm changes in mouse lungs arising due to smoking, and determine how these patterns would change after a period of smoking cessation. To this effect, we adapted an effective smoking mouse model which we have also used to measure the effects of early life smoke exposure on offspring [23]. Using this model, we identified 40 lung-specific CpGs which were differentially methylated following chronic smoke exposure, 32 of which had never been linked to smoking in the past. We also showed that chronic smoking causes alterations in mouse lung function that can last into adulthood, even after a long period of smoking cessation. These results are important, as identification of lung-specific DNAm patterns may provide more effective biomarkers for smoking, and may potentially be used to develop therapeutic interventions against the effects of chronic smoke exposure.

Prior to this study, much of what was known about smoking and DNAm was from studies conducted in blood in humans. Only a quarter of the sites we identified were associated with genes that had previously been connected to CS exposure in any tissue, and of those, there was existing evidence about DNAm differences specifically only at one gene, *Fkbp5* [48]. One study reported that smoking decreases *Fkbp5* DNAm in blood, which might be connected to dysregulation of the hypothalamic–pituitary–adrenal axis [48]. Our study replicated this finding, as we also observed a decrease in *Fkbp5* DNAm in mouse lungs our study, which might implicate the hypothalamic–pituitary–adrenal axis more broadly across tissues in the response to early life CS exposure.

Of the other genes with prior evidence of connection to CS exposure, the majority have genetic or transcriptomic connections. For example, one of our hits mapped to the Ataxin two (*Atxn2)* gene, expression of which has been linked to pancreatic cancer development in smokers [49]. The CpG we identified is located in an intragenic exon which is actively transcribed in mouse lungs, with high levels of H3K36me3 [50–52]. Generally, the function of DNAm at exons varies depending on where the exon is located [53–55], but most studies have discovered positive correlations between DNAm in intergenic exons and expression levels [53,56]. As our significant CpG is located in an intragenic exon, increased DNAm in smoking dams could signal an increase in *Atxn2* expression linked with an increased risk of malignancy. This is the first study associating smoking with altered DNAm at *Atxn2.*

We also found for the first time that heavy smoking resulted in a significant decrease in lung DNAm at Dopa decarboxylase (*Ddc*), a gene encoding a protein which catalyses the final steps of dopamine and serotonin biosynthesis [57]. Genetic variants in *Ddc*, especially at the introns [58], have been associated with smoking behaviour and nicotine dependency [58–60] in African-American and European-American populations. It therefore follows that alterations in DNAm at this gene as observed here could contribute to nicotine dependency in individuals who smoke. It is however important to note that genetic variants or genes alone do not determine susceptibility to addiction, and that the environment plays a major role [61,62]. In a Finnish twin study, it was discovered that the influence of genetics in adolescent smokers was decreased when the adolescents were highly monitored by their parents [63]. This emphasizes the need for more research to focus on understanding gene–environment interactions, as it could be crucial in deciphering the mechanism behind addiction.

The six other genes with prior evidence for association with smoking are summarized as follows: 1) Slit guidance ligand three (*Slit3*) whose expression has been associated with the development of nicotine preference in zebrafish [64], and which is down-regulated in human lung adenocarcinoma cell lines and tissues [65]. Expression levels of *SLIT3* are also significantly correlated with smoking history, as non-smokers with high expression of *SLIT3* had better prognosis among lung adenocarcinoma patients [65]; 2) Wingless-type MMTV integration site family, member 5A (*Wnt5a*) which is upregulated by tobacco smoke condensate in lung cancer cells [66], and in mouse and human lung tissues [67], and has been associated with the development of lung carcinogenesis in bronchial epithelial cells of smokers [68]; 3) Ankyrin repeat and SOCS box-containing 18 (*Asb18*) which shows differential expression levels in smokers [69]; 4) Nephronectin (*Npnt*), whose variants were shown to be involved in COPD-mediated airflow obstruction both in heavy smokers and non-smokers in a UK-based cohort [70]; 5) Membrane associated guanylate kinase, WW and PDZ domain containing 2 (*Magi2*) which shows higher expression in bronchial biopsies of heavy smokers with COPD [71]. *Magi2* polymorphisms may also influence nicotine dependence in smokers [72]; and 6) FK506 binding protein 5 (*Fkbp5*) which has been linked to DNAm changes in blood as discussed, but also modulates the effects of nicotine on the hypothalamic pituitary adrenal axis in female smokers [73].

Of the 32 novel sites identified in this study, two mapped to the promoter of *B3gnt3* (UDP-GlcNAc:betaGal beta-1,3-N-acetylglucosaminyltransferase 3) gene. Both CpGs, which are 32 bp apart, showed significantly higher DNAm in the lungs of dams exposed to CS compared to controls. This gene codes for a type II transmembrane protein which is involved in the biosynthesis of poly-N-acetyllactosamine chains, L-selectin ligand biosynthesis, lymphocyte homing and lymphocyte trafficking and recirculation [74,75]. One study reported increased *B3gnt3* expression levels corresponding to increased immune cell infiltration and correlated with poorer outcomes in patients with lung adenocarcinoma [76]. Other studies have also associated differential *B3gnt3* expression levels with development of many other types of cancers [77–81]. It therefore follows that increased DNAm in our two CpG sites, which are located in a weak promoter, could translate to decreased expression of the gene, consequently resulting in increased tolerance to and reduced sensitivity and response to foreign antigens by T cells. This is the first study associating smoking with altered DNAm at *B3gnt3.*

While there is a relative dearth of studies examining DNAm in the lungs, we found three other studies which directly measured effects of personal smoking on DNAm in the lungs. The first measured epigenome-wide DNAm in the blood of current and never-smokers. Next, using the identified significant CpGs from the epigenome-wide analysis, they measured DNAm in the lungs of current, ex- and never-smokers using pyrosequencing [14]. Out of 15 CpGs differentially methylated sites in the blood, only five replicated in the lungs, confirming that while these tissues share some similarities, not all blood-based findings will replicate in the lung [14]. Two of their five CpGs mapped to *AHRR*, and one corresponded to the human position we used to identify our mouse candidate *Ahrr* position (cg05575921). We did not observe DNAm changes in the lungs at *Ahrr*, but its poor conservation with the human genome in this region may limit comparability. In the second study, the effects of a) whole-body CS, b) the tobacco carcinogen 4-(methylnitrosamino)-1-(3-pyridyl)-1-butanone, and c) the inflammatory agent lipopolysaccharide were measured in mouse lungs using RRBS [20]. They found DNAm and hydroxymethylation differences across multiple differentially methylated regions [20]. While there were no overlapping differentially methylated sites between their results and ours, they identified 10 hydroxymethylated sites which corresponded to, or whose isoforms corresponded to 10 of our differentially methylated sites: *Strip1, Slit3, Megf6, Magi2, Atxn2, Sox10/Sox18, Wnt5b, Fkbp3, Asb13* and *Fbxo41/Fbxo45*. The microarrays we used here cannot distinguish between methylcytosine and hydroxymethylcytosine, and so it is possible that some of our findings are due to similar hydroxymethylcytosine changes. A third study exposed mice to 2 hours of CS twice daily, for 4 weeks consecutively in order to analyse the effect of smoking on lung DNAm. Using liquid hybridization capture-based bisulphite sequencing, they found that smoking altered DNAm patterns at numerous genes, most of which were involved in inflammation and in inflammatory injury in COPD, but due to limits on data availability it was not possible to determine whether specific findings overlapped with ours [21].

Gene ontology analysis on our list of significant genes revealed enrichment in multiple biological processes, with the most significant being involved in regulation of transmembrane receptor protein serine/threonine kinase signalling pathways. Components of CS, such as nicotine and the CS-specific carcinogen 4-(methylnitrosamino)-1-(3-pyridyl)-1-butanone, when bound to nicotinic-acetylcholine receptors, activate serine/threonine kinase Akt [82]. Activation of Akt resulted in increased phosphorylation of downstream molecules involved in controlling the cell cycle and protein translation, such as glycogen synthase kinase 3 (GSK-3) [82]. This in turn leads to cancer-like phenotypes such as loss of contact inhibition, desensitization to regulation by growth factors, decreased apoptosis, angiogenesis and increased cellular proliferation [82,83]. Since our current study identified an enrichment for genes involved in regulation of serine/threonine Akt pathways, it could support a predisposition to lung tumorigenesis in smoke-exposed mice.

Our candidate gene DNAm analysis also revealed interesting patterns. Our first candidate gene, *AHRR*, particularly locus cg05575921, has repeatedly shown decreased DNAm in human serum [84], whole and peripheral blood [9,85], saliva [86], monocytes [87] and alveolar macrophages [10] of current smokers compared to never-smokers. In the same vein, even offspring exposed to CS *in utero* have shown altered *AHRR* DNAm at the same locus compared to controls [28,88–90]. Compared to these studies however, our candidate gene analysis did not reveal significant differences in *Ahrr* DNAm or expression in blood or lungs at the corresponding mouse locus. This was interesting but not entirely surprising, since *Ahrr* is not well conserved between mice and humans at this region. Therefore, further mapping of DNAm in the *Ahrr* gene in mouse lung may reveal deeper insights.

At *Cyp1a1*, we found that smoking for 9 weeks caused a significant decrease in DNAm immediately after smoking, and that this pattern remained significant until 15 weeks after smoking cessation. However, *Cyp1a1* expression in mouse lungs after smoking cessation, though increased, was not significant, indicating that DNAm changes may last after expression differences have subsided. *Cyp1a1* belongs to the cytochrome p450 enzyme subclass, and is involved in drug/xenobiotic metabolism [91–93]. Therefore, reduced DNAm of *Cyp1a1* in CS-exposed mice as observed in this study could indicate induction of the enzyme to aid detoxification of CS components. These results mimic reduced *Cyp1a1* DNAm and increased expression patterns observed in the lungs [94], adipose tissue [95], prostate cancer tissues [96], buccal cells [19] and blood [19] of humans exposed to CS in previous studies. Even though none of the *Cyp1a1* probes in the mouse methylation microarray was among our 40 significant CpGs, we showed that 3 of the 9 *Cyp1a1* probes have large differences in DNAm. Our inability to detect these positions (and likely some other CpGs) as significant is likely due to our small sample size. So, while these 3 probes met our strict effect size cut-off of 0.05, they did not meet the *p* value cut-off. Future studies with larger sample sizes would be helpful in generating more precise measurements.

In addition to molecular alterations, we also assessed the effects of chronic smoking on physiological outcomes in the form of lung function. We observed that the changes in lung function immediately after smoking for 9 weeks were small and not significant. However, 15 weeks after smoking cessation, lung function in CS-exposed dams had significantly declined, as evidenced by increased airway responsiveness to methacholine. One reason why lung function worsens over time in our smoke-exposed mice may be due to ageing. Past research indicates that lung function declines with age due to structural and immunological factors which impair gas exchange and increase susceptibility to infections [97–99]. Other studies report that smoking exacerbates this lung function decline, even in ex-smokers [100–103]. This trend of worsening/decline in lung function is a characteristic of COPD [104,105]. In line with our finding of declining lung function even with smoking cessation, in a large multi-ethnic pooled cohort study [103], smoking cessation slightly improved lung function, but these ‘improvements’ still resulted in lung function similar to light/low-intensity current smokers. In fact, smoking cessation does not completely restore lung function to the levels of never-smokers [103]. This pattern of lung function decline despite smoking cessation may be due to sustained dysregulation of epigenetic patterns [106–108], immune responses [109–111] and airway hyperresponsiveness [112].

This study represents an important contribution to our understanding of the impacts of CS on the lungs, but is not without limitations. The first limitation is our small sample size for the EWAS and hence, low statistical power in identifying small DNAm differences. We also could not distinguish between hydroxymethylcytosine and methylcytosine, meaning that some of our differential DNAm findings may be due to changes in hydroxymethylcytosine. It is also worth noting that whole lungs were used in this study, making it impossible to determine whether differences in cell composition are masking some of our findings. However, we took two approaches to mitigate this problem. First, we lavaged the lungs prior to analysis to remove immune cells which we know to be different between CS and control animals, making it unlikely that immune cells are responsible for our observed results. To further support this, we found that *Cyp1a1* DNAm in the blood of adult female control mice (Figure 4(a)) is higher than *Cyp1a1* DNAm in lungs of control mice (Figure 4(d)); therefore, infiltration of immune cells from blood to lungs is unlikely to cause the decrease in lung *Cyp1a1* DNAm in the present study. Second, we used surrogate variables to control for variance likely associated with cell type. This approach removes the effects of cell type such that further work will be required to determine which lung cell types exhibit the observed DNAm differences.

In summary, we have shown here that chronic smoking alters DNAm patterns in mouse lungs at multiple CpGs, some which have never been associated with smoking in the past. These CpGs map to genes implicated in tumorigenesis and metastasis, neurodegenerative disorders, nicotine dependency and immune cell responses. Moreover, lung function defects persist up to 15 weeks after smoking cessation. The fact that 75% of the significant CpGs reported here have never been linked to CS in the past, even in studies which investigated effects of CS in the blood, emphasizes the need for more studies to be done on the lungs, as it clearly has different responses than other tissues. This research is one part of a much larger study to investigate the effects of early life CS exposure on offspring DNAm patterns. Therefore, the results presented here will hopefully also shed more light on some of the patterns we observe in offspring exposed to CS *in utero*.

## Supplementary Material

Figure S4_dam paper.tif

Table S1_Sample information.xlsx

Figure S2_Cyp1a1_Gviz_dam paper.tif

Figure S3_dam paper.tif

Figure S1_dam paper.tif

## Data Availability

The data supporting the findings from this study are available from the corresponding author, M.J.J., upon request.
