# Supplementary figures and images for: Novel DNA methylation changes in mouse lungs associated with chronic smoking
Source: Epigenetics. 2024 Mar 4;19(1):2322386. doi: 10.1080/15592294.2024.2322386 (PMC10913724; doi:10.1080/15592294.2024.2322386)

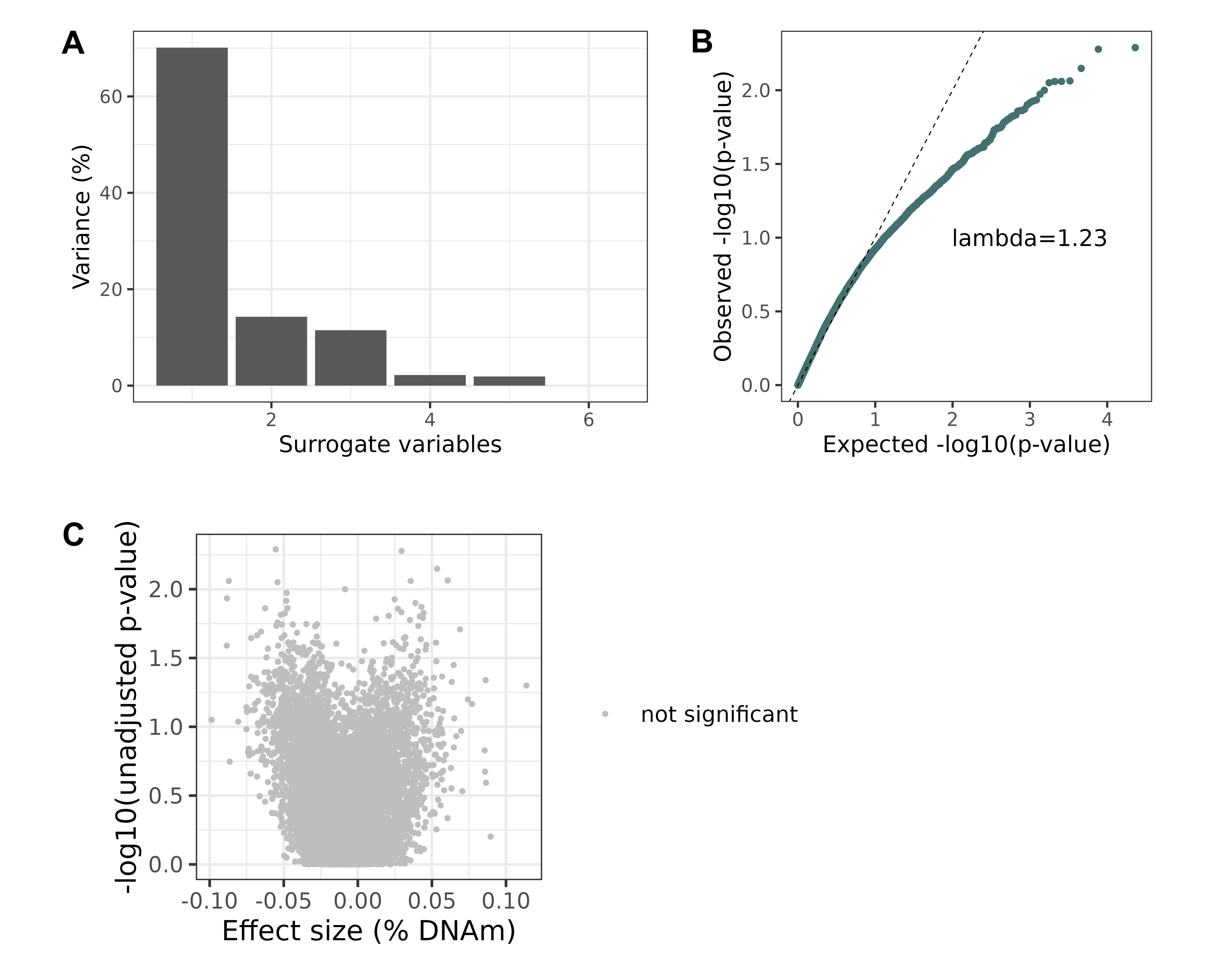

Supplement: Figure S4_dam paper.tif [file KEPI_A_2322386_SM8174.tif]

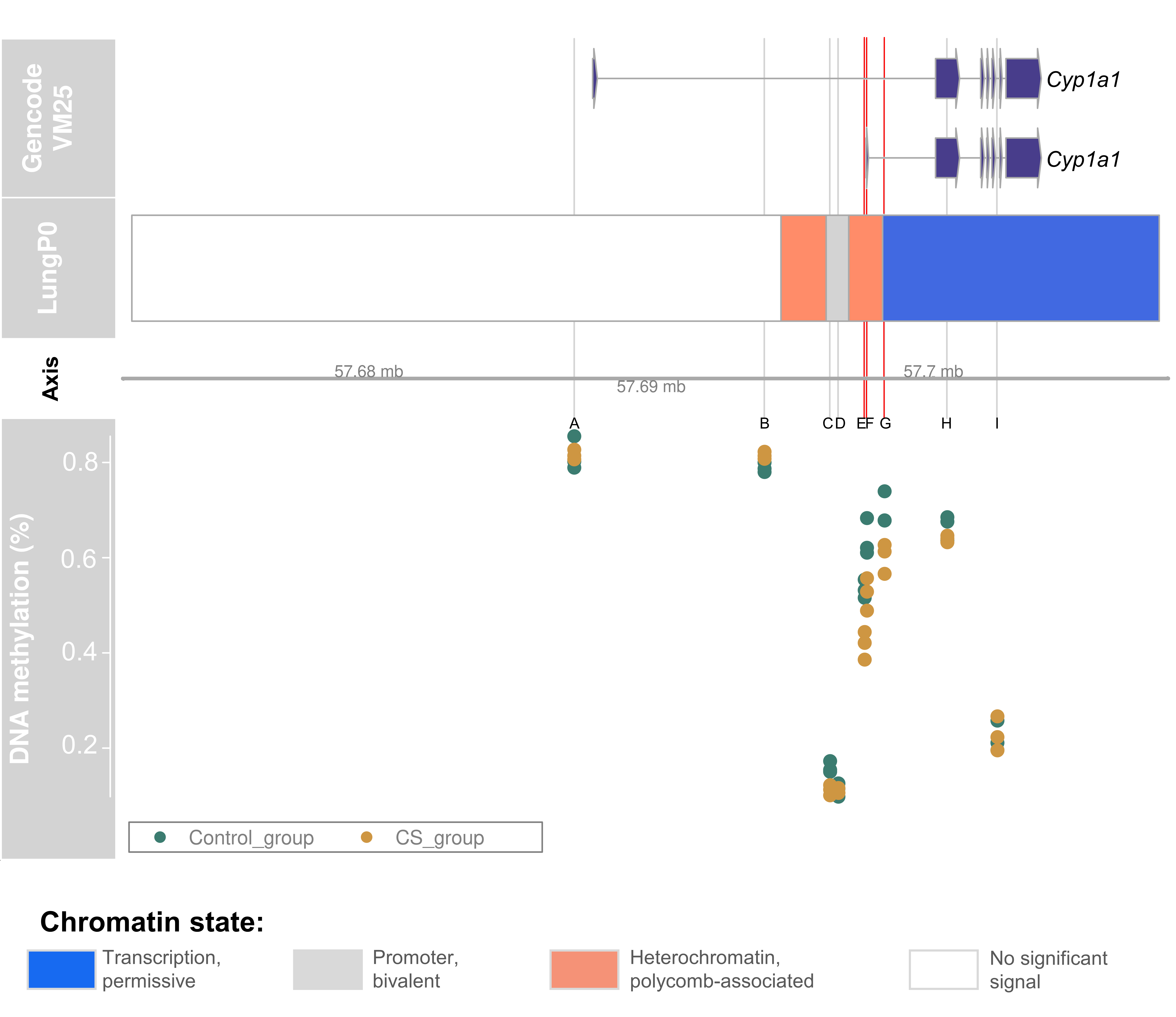

Supplement: Figure S2_Cyp1a1_Gviz_dam paper.tif [file KEPI_A_2322386_SM8172.tif]

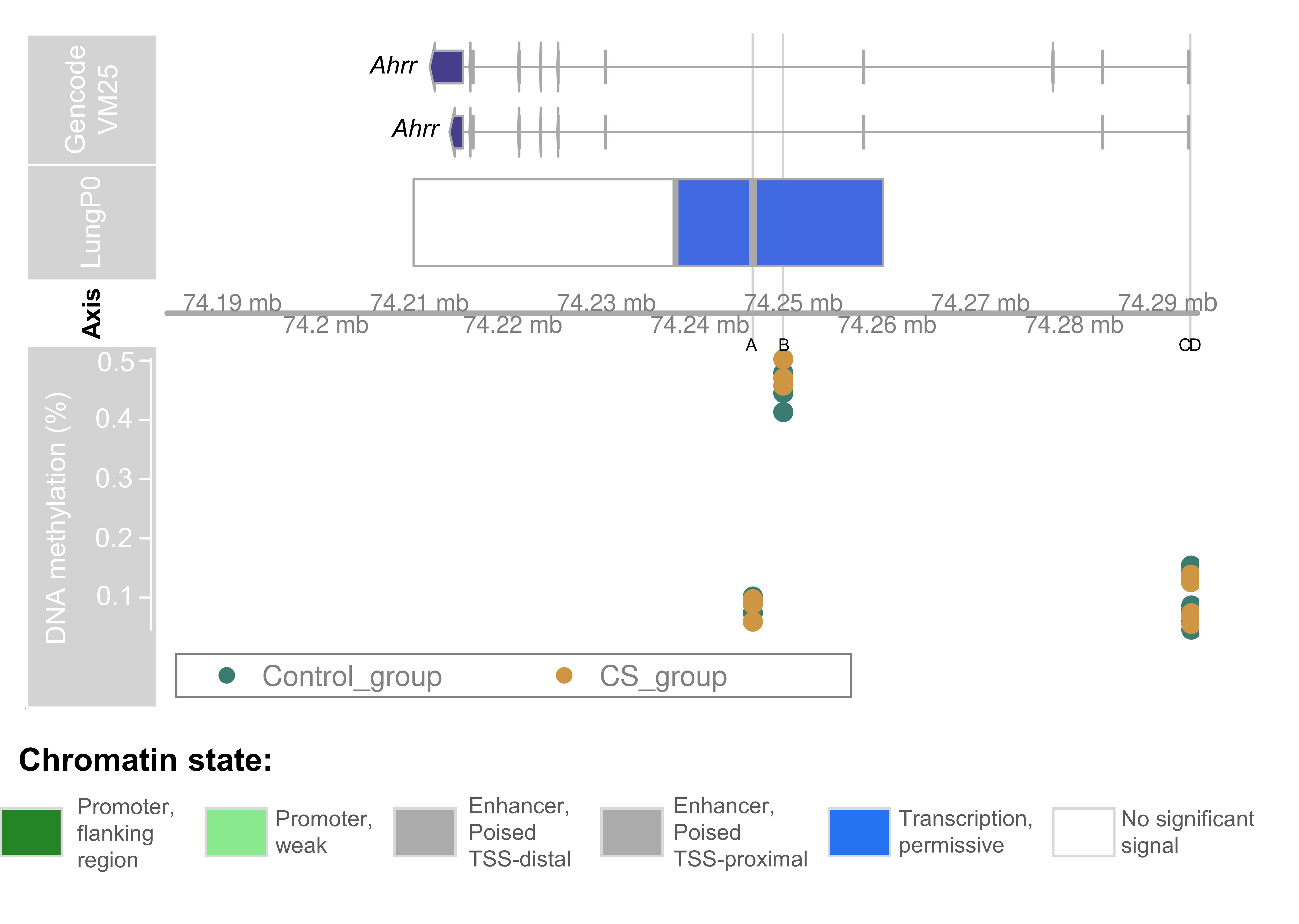

Supplement: Figure S3_dam paper.tif [file KEPI_A_2322386_SM8171.tif]

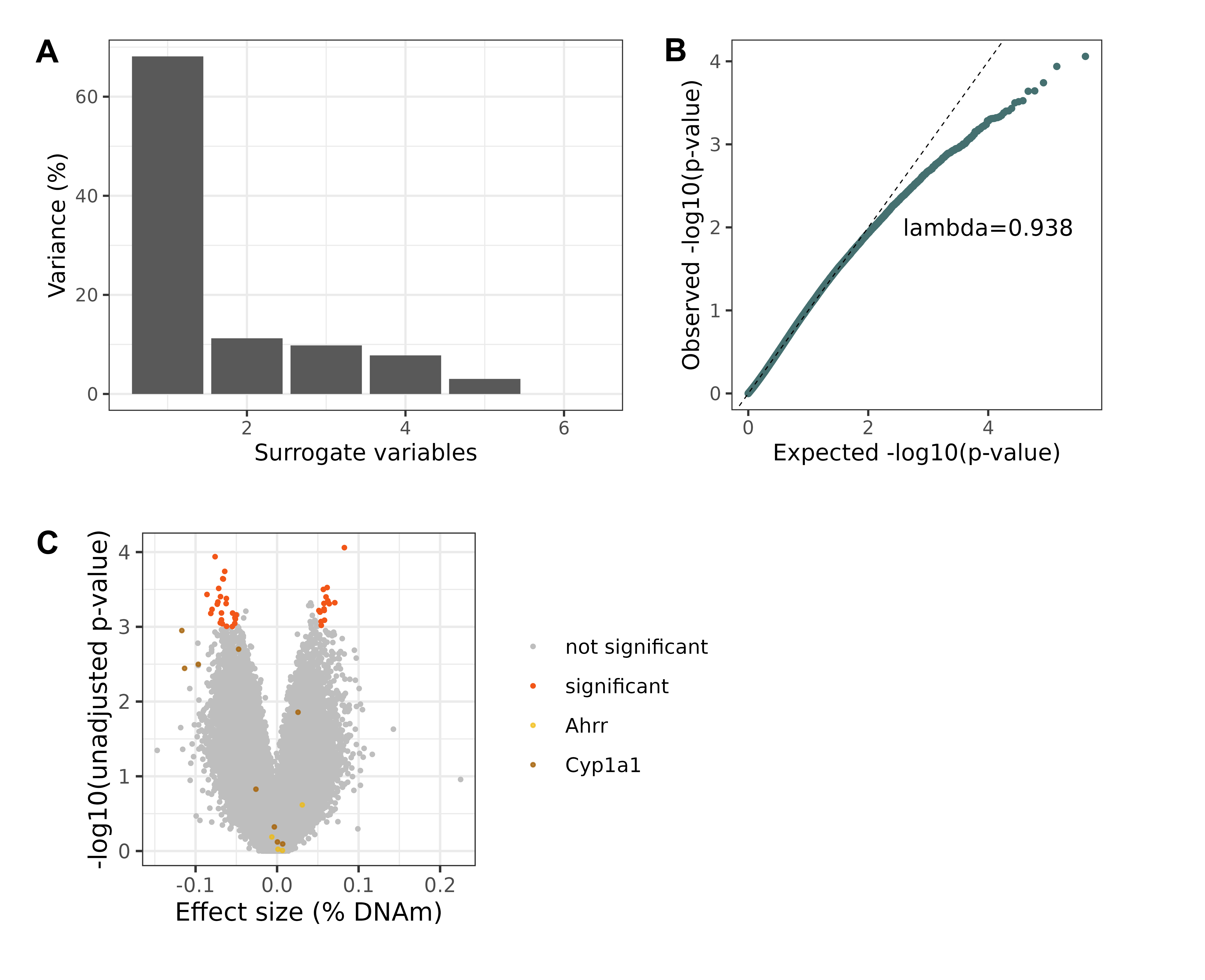

Supplement: Figure S1_dam paper.tif [file KEPI_A_2322386_SM8170.tif]
